# Supplementary material for: RNA chaperones buffer deleterious mutations in E. coli
Source: eLife. 2015 Mar 25;4:e04745. doi: 10.7554/eLife.04745 (PMC4402597; doi:10.7554/eLife.04745)
Supplement: Supplementary file 4. — List of Escherichia coli strains used in this study. DOI: http://dx.doi.org/10.7554/eLife.04745.019 [file elife04745s004.docx]

**Supplementary file 4.** List of *E. coli* strains used in this study*.

| Strain | Characteristics | Source, Reference or Construction |
| --- | --- | --- |
| MG1655 |  | Lab stock |
| ΔaraA | *E.coli* MG1655Δ*araA* ::Kan/Ara- | NBRP |
| MR01 | MG1655 Δ*araA*::Kan/Ara- | Transduction with P1(Δ*araA* ::Kan) |
| MR02 | MR01 pCA24N::Cam/Ara- | Transformation |
| MR03 | MG1655 pCA24N-*cspA*::Cam/Ara+ | Transformation |
| MR04 | MG1655 pCA24N-*rhlB*::Cam/Ara+ | Transformation |
| MR05 | MG1655 pCA24N-*srmB*::Cam/Ara+ | Transformation |
| MR06 | MG1655 pCA24N-*csdA*::Cam/Ara+ | Transformation |
| ΔmutH | MG1655 Δ*mutH*::Kan /Ara+ | Keio collection |
| MR07 | Δ*mutH* /Ara- | Selection on TA plates |
| MR08 | MR07 pCA24N::Cam/Ara- | Transformation |
| MR09 | Δ*mutH* pCA24N-*cspA*::Cam/Ara+ | Transformation |
| MR10 | Δ*mutH* pCA24N-*rhlB*::Cam/Ara+ | Transformation |
| MR11 | Δ*mutH* pCA24N-*srmB*::Cam/Ara+ | Transformation |
| MR12 | Δ*mutH* pCA24N-*csdA*::Cam/Ara+ | Transformation |
| REL607 | *E.coli* B/Ara+ | (1) |
| REL606 | *E.coli* B /Ara- | (1) |
| MR13 | REL606 pCA24N::Cam/Ara- | Transformation |
| MR14 | REL607 pCA24N-*cspA*::Cam/Ara+ | Transformation |
| MR15 | REL607 pCA24N-*rhlB*::Cam/Ara+ | Transformation |
| MR16 | REL607 pCA24N-*srmB*::Cam/Ara+ | Transformation |
| MR17 | REL607 pCA24N-*csdA*::Cam/Ara+ | Transformation |
| REL 10953 | Ara+ | (1) |
| MR18 | REL 10953/Ara- | Selection on TA plates |
| MR19 | MR18 pCA24N::Cam/Ara- | Transformation |
| MR20 | REL 10953 pCA24N-*cspA*::Cam/Ara+ |  |
| MR21 | REL 10953 pCA24N-*rhlB*::Cam/Ara+ | Transformation |
| MR22 | REL 10953 pCA24N-*srmB*::Cam/Ara+ | Transformation |
| MR23 | REL 10953 pCA24N-*csdA*::Cam/Ara+ | Transformation |
| *de novo* ΔmutH | MG1655 Δ*mutH*::Kan /Ara+ | Transduction with P1 (Δ*mutH*::Kan) |
| MR24 | *de novo* Δ*mutH*/Ara- | Selection on TA plates |
| MR25 | MR24 pCA24N::Cam/Ara- | Transformation |
| MR26 | *de novo* Δ*mutH* pCA24N-*cspA*::Cam/Ara+ | Transformation |
| MR27 | *de novo* Δ*mutH* pCA24N-*rhlB*::Cam/Ara+ | Transformation |
| MR28 | *de novo* Δ*mutH* pCA24N-*srmB*::Cam/Ara+ | Transformation |
| MR29 | *de novo* Δ*mutH* pCA24N-*csdA*::Cam/Ara+ | Transformation |
| REL 8602A | Ara+ | (1) |
| MR30 | REL 8602A/Ara- | Selection on TA plates |
| MR31 | MR30 pCA24N::Cam/Ara- | Transformation |
| MR32 | REL 8602A pCA24N-*cspA*::Cam/Ara+ | Transformation |
| MR33 | REL 8602A pCA24N-*rhlB*::Cam/Ara+ | Transformation |
| MR34 | REL 8602A pCA24N-*srmB*::Cam/Ara+ | Transformation |
| MR35 | REL 8602A pCA24N-*csdA*::Cam/Ara+ | Transformation |
| MR36 | MR01 pJ444-01::Amp/Ara- | Transformation |
| MR37 | MG1655 pJ444-01-*cspA*::Amp F20L/Ara+ | Transformation |
| MR38 | MG1655 pJ444-01-*rhlB*::Amp E166K/Ara+ | Transformation |
| MR39 | MG1655 pJ444-01-*srmB*::Amp E158K/Ara+ | Transformation |
| MR40 | MG1655 pJ444-01-*csdA*::Amp E157K/Ara+ | Transformation |
| MR41 | MG1655 Δ*mutH*::Cam /Ara+ | Transduction with P1 (Δ*mutH*::Cam) |
| MR42 | MR41/Ara- | Selection on TA plates |
| MR43 | MR42 pJ441-01::Kan/Ara- | Transformation |
| MR44 | MR41 pJ441-01-*cspA*::Kan F20L /Ara+ | Transformation |
| MR45 | MR41 pJ441-01-*rhlB*::Kan E166K/Ara+ | Transformation |
| MR46 | MR41 pJ441-01-*srmB*::Kan E158K/Ara+ | Transformation |
| MR47 | MR41 pJ441-01-*csdA*::Kan E157K/Ara+ | Transformation |
| MR48 | REL606 pJ441-01::Kan/Ara- | Transformation |
| MR49 | REL607 pJ441-01-*cspA*::Kan F20L/Ara+ | Transformation |
| MR50 | REL607 pJ441-01-*rhlB*::Kan E166K/Ara+ | Transformation |
| MR51 | REL607 pJ441-01-*srmB*::Kan E158K/Ara+ | Transformation |
| MR52 | REL607 pJ441-01-*csdA*::Kan E157K/Ara+ | Transformation |
| MR53 | MR18 pJ441-01::Kan/Ara- | Transformation |
| MR54 | REL 10953 pJ441-01-*cspA*::Kan F20L /Ara+ | Transformation |
| MR55 | REL 10953 pJ441-01-*rhlB*::Kan E166K/Ara+ | Transformation |
| MR56 | REL 10953 pJ441-01-*srmB*::Kan E158K/Ara+ | Transformation |
| MR57 | REL 10953 pJ441-01-*csdA*::Kan E157K/Ara+ | Transformation |

*strains carrying individual point mutants are derived as indicated in the Methods section.

1. Wiser, M.J., Ribeck, N. &Lenski, R.E. Long-Term Dynamics of Adaptation in Asexual Populations. *Science* **342**,1364-1367 (2013).
